# Detection and localization of caries and hypomineralization on dental photographs with a vision transformer model

**DOI:** 10.1038/s41746-023-00944-2

**Published:** 2023-10-25

**Authors:** Marco Felsch, Ole Meyer, Anne Schlickenrieder, Paula Engels, Jule Schönewolf, Felicitas Zöllner, Roswitha Heinrich-Weltzien, Marc Hesenius, Reinhard Hickel, Volker Gruhn, Jan Kühnisch

**Affiliations:** 1 Department of Conservative Dentistry and Periodontology, School of Dentistry, Ludwig-Maximilians University of Munich, Munich, Germany; 2 Institute for Software Engineering, University of Duisburg-Essen, Essen, Germany; 3 Department of Orthodontics, Section of Preventive and Paediatric Dentistry, University Hospital Jena, Jena, Germany

**Keywords:** Oral diseases, Dentistry, Dental caries, Diagnosis

## Abstract

Caries and molar-incisor hypomineralization (MIH) are among the most prevalent diseases worldwide and need to be reliably diagnosed. The use of dental photographs and artificial intelligence (AI) methods may potentially contribute to realizing accurate and automated diagnostic visual examinations in the future. Therefore, the present study aimed to develop an AI-based algorithm that can detect, classify and localize caries and MIH. This study included an image set of 18,179 anonymous photographs. Pixelwise image labeling was achieved by trained and calibrated annotators using the Computer Vision Annotation Tool (CVAT). All annotations were made according to standard methods and were independently checked by an experienced dentist. The entire image set was divided into training (*N* = 16,679), validation (*N* = 500) and test sets (*N* = 1000). The AI-based algorithm was trained and finetuned over 250 epochs by using image augmentation and adapting a vision transformer network (SegFormer-B5). Statistics included the determination of the intersection over union (IoU), average precision (AP) and accuracy (ACC). The overall diagnostic performance in terms of IoU, AP and ACC were 0.959, 0.977 and 0.978 for the finetuned model, respectively. The corresponding data for the most relevant caries classes of non-cavitations (0.630, 0.813 and 0.990) and dentin cavities (0.692, 0.830, and 0.997) were found to be high. MIH-related demarcated opacity (0.672, 0.827, and 0.993) and atypical restoration (0.829, 0.902, and 0.999) showed similar results. Here, we report that the model achieves excellent precision for pixelwise detection and localization of caries and MIH. Nevertheless, the model needs to be further improved and externally validated.

## Introduction

Caries is among the most prevalent non-communicable diseases in all age groups worldwide^1,2^, and developmental disorders such as molar-incisor hypomineralization (MIH)—synonymously named “chalky teeth”—are of additional clinical relevance, especially in younger populations^3^. Both entities need to be reliably diagnosed by dental professionals. Here, a visual examination (VE) must be recognized as the method of choice for caries and MIH detection due to its simplicity, rapidness, and documented validity^4–9^. However, when considering the documented diagnostic variability between different dentists or work groups^5,6^, it can be argued that the reliability of VE can be improved and should optimally be as objective as possible. Following this aim, the use of teeth photographs—a digital and machine-readable equivalent to a clinical examination—and artificial intelligence (AI) methods may potentially contribute to accurate diagnostic evaluations in the future. Recently, a few study groups have used and evaluated convolutional neural networks (CNNs) with digital photographs for the detection of caries^10–12^ and MIH^13,14^. All studies proved the concept of using AI-based methods for dental photographs, and promising results were published. While a publicly accessible model would enable an independent evaluation by other research groups, no such model has been introduced thus far. Most recently, vision transformer networks were introduced as an alternative to established CNNs for various image recognition tasks^15^. Considering their computational efficiency and accuracy, it might be possible that transformers may outperform current CNN standards in the future. AI-based solutions for detecting pathologies, including caries and MIH, should optimally be based on this new technology, which has rarely been applied in medicine and dentistry until now^16–19^.

Therefore, the present study first aims to develop a transformer-based model to achieve precise and simultaneous pixelwise detection and localization of relevant caries and MIH classes from dental photographs. Second, it is hypothesized that the model could achieve an accuracy of at least 98% and an average precision of 0.5 for the detection and localization of caries and MIH classes. The final study aim is to make the AI-based model publicly accessible as a web application.

## Results

### Data set

In the complete image set, 34,710 pathological findings belonging to the caries (*N* = 26,360) and MIH (*N* = 8350) entities were detected and classified. Non-cavitated caries lesions and dentin cavities were found to be the most frequent caries classifications. Hypomineralized teeth were predominantly characterized by demarcated opacities and enamel disintegrations. Detailed distributions among classifications in relation to the training, validation and test sets can be observed in Table 1.

**Table 1:** Overview of the included pixelwise annotations for the training set (*N* = 16,679 images), validation set (*N* = 500 images), and the independent test set (*N* = 1000 images).

Diagnostic category	Number of annotations			
	Training set	Validation set	Test set	Total
Caries				
Non-cavitation	16,185	501	1058	17,744
Grayish translucency	954	29	67	1050
Enamel breakdown	1001	33	70	1104
Dentin cavity	5046	155	316	5517
Fully destructed tooth	868	27	50	945
Molar–incisor hypomineralization (chalky teeth)				
Demarcated opacity	5817	180	329	6326
Enamel disintegration	1143	35	69	1247
Atypical restoration	721	22	34	777
Total	31,735	982	1993	34,710

### Model performance

**Fig. 1:**
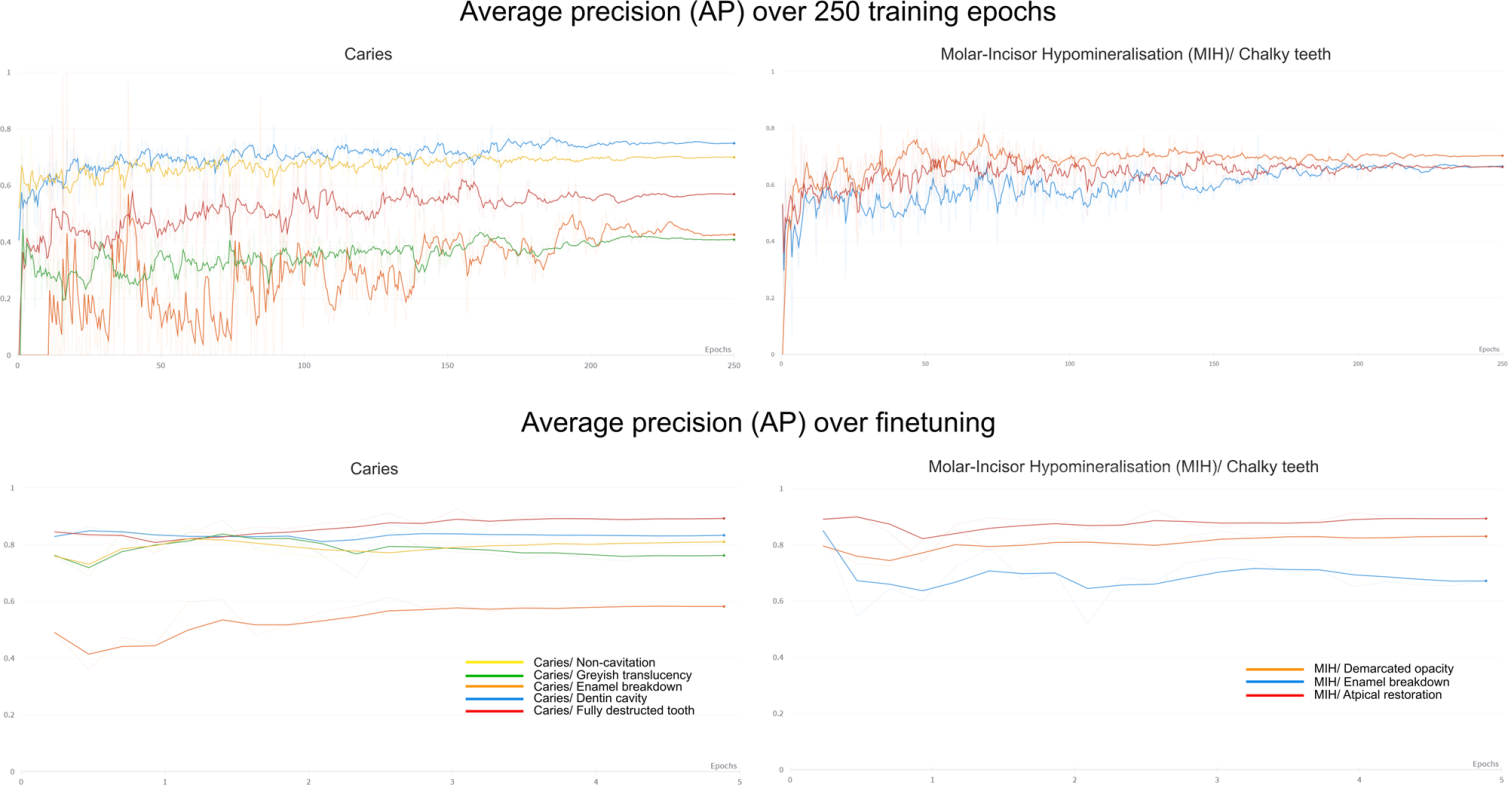
Average precision (AP) in relation to the training progress for the caries and MIH categories. All lines in graphs are plotted over 250 epochs.

The highest pixel numbers for caries were documented for non-cavitated lesions and dentin cavities (Table 2). In contrast, the pixel counts for grayish translucencies and enamel breakdowns were lower by factors of ~30 and ~50, respectively (Table 2). In the MIH entity, demarcated opacities were labeled most often, followed by atypical restorations and enamel disintegrations (Table 2). The diagnostic performance in terms of F1-score, IoU, AP and ACC for each class can also be observed in Table 2. Notably, even after baseline training, most of the IoU values were above 0.4 (Table 2), except caries-related grayish translucencies (IoU = 0.210) and enamel breakdowns (IoU = 0.088). The IoU values increased up to ~0.8 after finetuning. However, caries-associated enamel breakdowns (IoU = 0.352) and enamel disintegrations due to MIH (IoU = 0.507) remained lower than all others (Table 2). The model’s overall IoU value was 0.959 after finetuning. When considering the AP, the same pattern emerged (Table 2 and Fig. 1). After baseline training, the AP values ranged between 0.420 and 0.751 for the caries classes and between 0.657 and 0.704 for the MIH classes. The model performance once again increased after finetuning for caries (0.588–0.882) and MIH (0.669–0.902); the overall AP reached 0.977. The ACC on pixel level was found to be constantly high throughout baseline training as well as finetuning and exceeded—with one exception—values above 0.99. The overall ACC was 0.978 after finetuning (Table 2).

**Table 2:** Diagnostic performance of the transformer-based model on a pixel level after 250 training epochs and additional finetuning.

Diagnostic category	Total pixel number (*N* × 10^6^)	F1	IoU	Average precision	Accuracy
*Diagnostic performance after 250 training epochs (baseline training)*					
Caries					
Non-cavitation	6.096	0.595	0.423	0.683	0.983
Grayish translucency	0.260	0.347	0.210	0.420	0.999
Enamel breakdown	0.139	0.161	0.088	0.468	0.999
Dentin cavity	2.713	0.763	0.617	0.751	0.995
Fully destructed tooth	0.922	0.630	0.460	0.542	0.997
Molar–incisor hypomineralization (chalky teeth)					
Demarcated opacity	3.715	0.586	0.423	0.657	0.990
Enamel disintegration	0.688	0.604	0.433	0.674	0.998
Atypical restoration	1.552	0.669	0.503	0.704	0.996
None	246.057	0.984	0.969	0.980	0.970
Total	262.142	0.962	0.937	0.961	0.964
*Diagnostic performance after 250 training epochs + finetuning*					
Caries					
Non-cavitation	6.386	0.773	0.630	0.813	0.990
Grayish translucency	0.292	0.746	0.595	0.743	0.999
Enamel breakdown	0.136	0.521	0.352	0.588	0.999
Dentin cavity	2.471	0.818	0.692	0.830	0.997
Fully destructed tooth	1.674	0.881	0.787	0.882	0.999
Molar–incisor hypomineralization (chalky teeth)					
Demarcated opacity	4.758	0.804	0.672	0.827	0.993
Enamel disintegration	0.322	0.673	0.507	0.669	0.999
Atypical restoration	1.566	0.906	0.829	0.902	0.999
None	244.539	0.990	0.979	0.988	0.981
Total	262.144	0.977	0.959	0.977	0.978

In addition to the pixelwise analysis (Table 2), Table 3 summarizes the model performance for caries and MIH detection on an image level. The overall diagnostic ACC values were found to be high, with numbers above 95%. SE and SP ranged between ~80% and ~100%. Only in the case of caries-related enamel breakdowns was low SE documented (Table 3).

**Table 3:** Overview of the model performance per image in relation to the main diagnostic classes using the independent test set (*N* = 1000 images).

	True positives (TP)		True negatives (TN)		False positives (FP)		False negatives (FN)		Diagnostic performance				
	*N*	%	*N*	%	*N*	%	*N*	%	ACC	SE	SP	NPV	PPV
*Diagnostic performance after 250 training epochs (baseline training)*													
Caries													
Non-cavitation	372	37.2	498	49.8	62	6.2	68	6.8	87.0	84.6	88.9	88.0	85.7
Grayish translucency	26	26.0	952	95.2	10	1.0	12	1.2	97.8	68.4	99.0	98.8	72.2
Enamel breakdown	13	1.3	961	96.1	1	0.1	25	2.5	97.4	34.2	99.9	97.5	92.9
Dentin cavity	155	15.5	791	79.1	23	2.3	31	3.1	94.6	83.3	97.2	96.2	87.1
Fully destructed tooth	40	4.0	949	94.1	5	0.5	6	0.6	98.9	87.0	99.5	99.4	88.9
Molar–incisor hypomineralization (chalky teeth)													
Demarcated opacity	148	14.8	807	80.7	27	2.7	18	1.8	95.5	89.2	96.8	97.8	84.6
Enamel disintegration	38	38.0	954	95.4	4	0.4	4	0.4	99.2	90.5	99.6	99.6	90.5
Atypical restoration	35	3.5	954	95.4	5	0.5	6	0.6	98.9	85.4	99.5	99.4	87.5
*Diagnostic performance after 250 training epochs + finetuning*													
Caries													
Non-cavitation	389	38.9	512	51.2	5	0.5	5	0.5	90.1	88.4	91.4	90.9	89.0
Grayish translucency	31	31.0	959	95.9	3	0.3	7	0.7	99.0	81.6	99.7	99.3	91.2
Enamel breakdown	14	1.4	954	95.4	8	0.8	24	2.4	96.8	36.8	99.2	97.5	63.6
Dentin cavity	163	16.3	796	79.6	18	1.8	23	2.3	95.9	87.6	97.8	97.2	90.1
Fully destructed tooth	41	4.1	949	94.9	5	0.5	5	0.5	99.0	89.1	99.5	99.5	89.1
Molar–incisor hypomineralization (chalky teeth)													
Demarcated opacity	156	15.6	813	81.3	21	2.1	10	1.0	96.9	94.0	97.6	98.8	88.1
Enamel disintegration	33	3.3	955	95.5	4	0.4	8	0.8	98.8	80.5	99.6	99.2	89.2
Atypical restoration	38	3.8	957	95.7	1	0.1	4	0.4	99.5	90.5	99.9	99.6	97.5

## Discussion

**Fig. 2:**
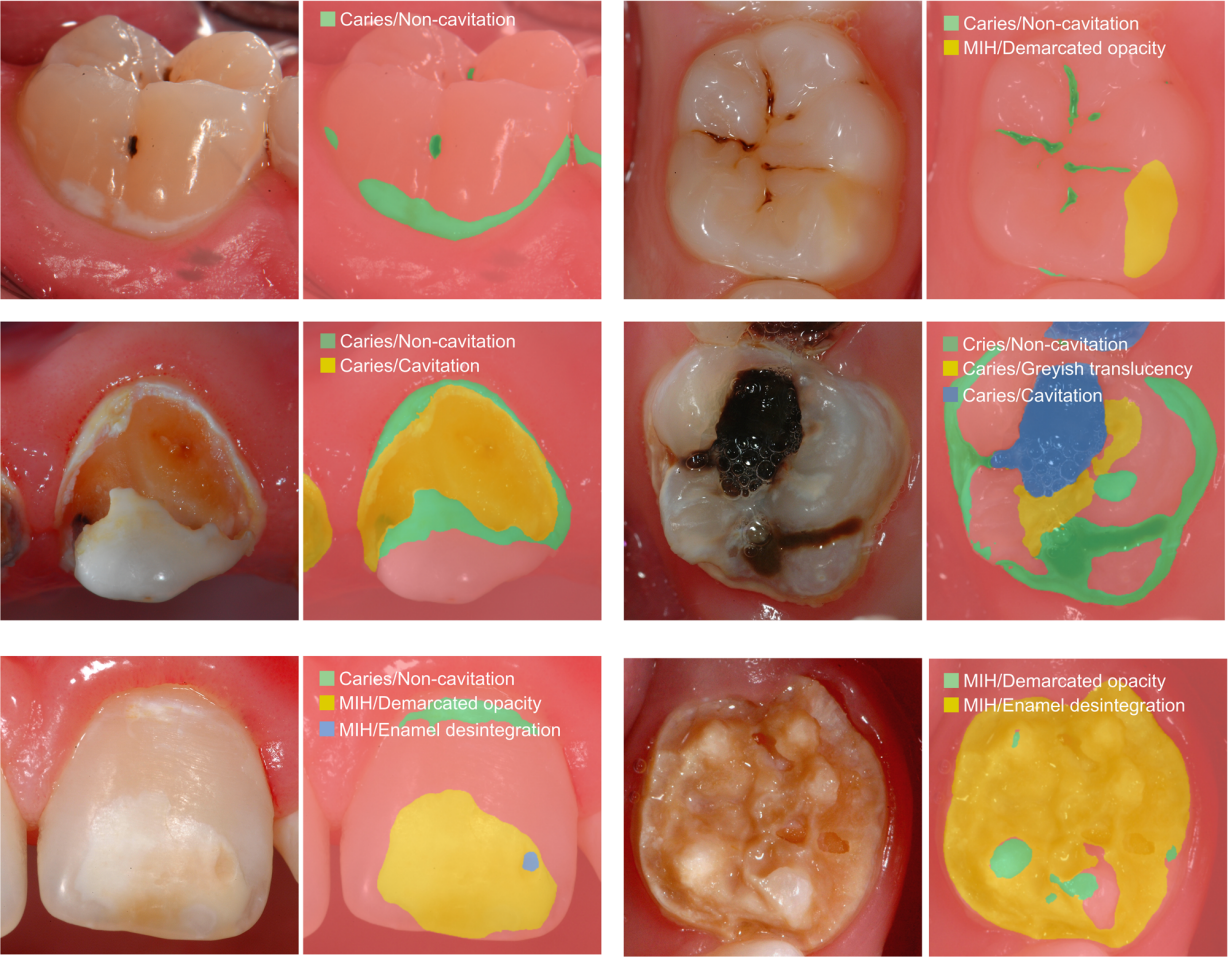
Examples of clinical images and the corresponding outputs by the segmentation model. The description and the corresponding false coloured segments indicate the diagnostic category.

This study developed and evaluated an AI-based diagnostic model for the detection, classification, and localization of caries as well as MIH in professionally captured clinical photographs of teeth. Furthermore, the model was made openly accessible as a web application (http://demo.dental-ai.de). In particular, the use of precise object labeling in a large image set and pixelwise image analysis utilizing a transformer network with a segmentation head resulted in a model that can simultaneously identify different pathologies, including subscores, from dental photographs (Fig. 2).

The comparison and interpretation of the shown data for pixelwise analysis (Table 2 and Fig. 1) is limited at the moment, simply due to the lack of technically comparable projects in dentistry. However, the following discussion should give an overview of the recent state of knowledge. In general, the transformer model achieved an overall ACC value of 0.978 at the pixel level, and in the majority of the included diagnostic categories, an ACC value >0.99 was reached. In the case of non-cavitated caries, the ACC was 0.99. It can be concluded that the ACC was very high, which is in line with the available literature evaluating transformers^16–18^, and finally, the initially formulated goal was reached. When comparing the documented ACC values (>95%) from the image-related analysis (Table 3) to those from previously published data using CNNs, ACC values of approximately 90% were achieved for caries^10–12^ and MIH detection^13,14^. This comparison indicates that the use of exact annotations and a powerful transformer network, as well as other improvements such as pixelwise analysis and the inclusion of commonly used caries and MIH categories, may surpass CNN-based algorithms in terms of diagnostic performance. Nevertheless, it should be noted that misclassification is possible and might predominantly be linked to lesions of smaller size.

In terms of the AP, the anticipated value of 0.5 was even exceeded after finetuning, with individual values of up to 0.902 (Table 2 and Fig. 1). These values match those of other current studies in medicine^17,18^ and dentistry^16^ for radiographs. Interestingly, the AP and IoU values may be influenced by the overall pixel number and depend further on the number of annotations. In other words, all high-frequency categories with a large pixel quantity, e.g., non-cavitated caries, dentin cavity caries or MIH-related opacities (Tables 1 and 2), were found to be associated with higher AP and IoU values. In contrast, less-frequent categories with small-sized lesions, e.g., caries-related grayish translucencies and enamel breakdowns as well as MIH-related enamel disintegrations (Tables 1 and 2), were generally linked to lower AP and IoU values. This finding might be explained by the small-sized lesions and possible edge inaccuracies that potentially occur during labeling. Considering the latter aspect, it is inevitable that the manually drawn labels around any pathology will also contain pixels of sound dental hard tissue. This may confuse the model during training and affect its accuracy in general, possibly more severely in cases of less frequent and small-sized dental defects. To overcome this issue and further improve diagnostic performance, a continuous increase in the number of images, especially those of the previously mentioned pathologies, should be carried out. Consequently, future research is needed to address this issue.

In medicine, transformer-based AI algorithms have predominantly been used for language or text recognition and processing tasks^19^. Meanwhile, they have also been used for object detection^15–18^. The use of a transformer network with a segmentation head (following the SegFormer architecture) has the advantage that diagnostic decisions of the model can be made on the pixel level. Classification and localization are thus unified in one step, and a segmentation map, which may contain multiple diagnoses for the image at once and allows size and location estimation, is generated. Due to the available hardware resources, it was possible to process all images with an appropriate resolution, which probably contributed to the precision of the developed algorithm.

**Fig. 3:**
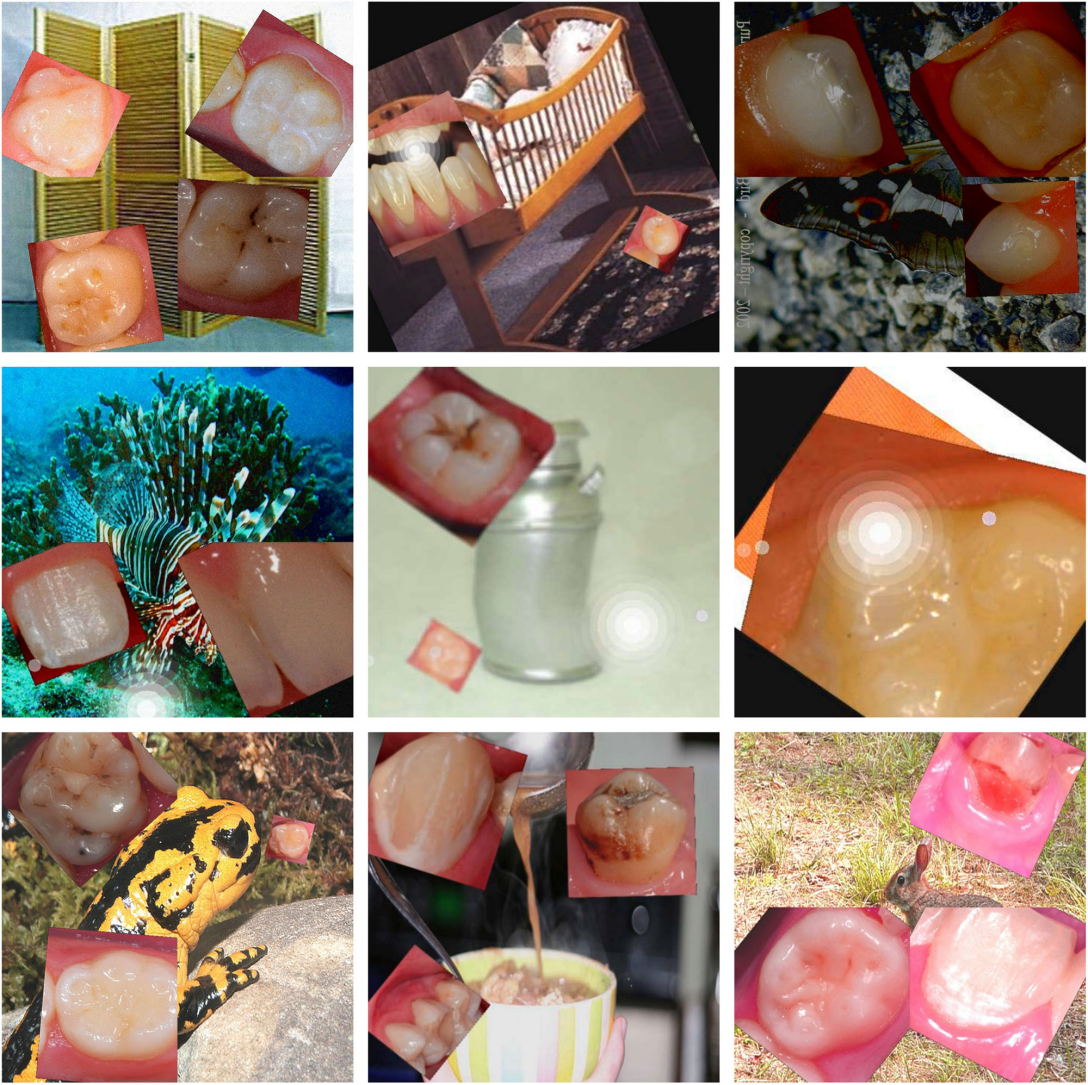
Examples of augmented images that were continuously generated during the training process. More than four million augmented images were used to train the vision transformer-based model over 250 epochs.

This study has several strengths and limitations. The sizeable number of dental photographs (*N* = 18,719) combined with the fact that all images were individually annotated pixelwise and counterchecked by trained and calibrated dentists according to widely accepted classification systems must be highlighted as fundamental features. The utilized image augmentation procedures may have contributed substantially to the fact that there was a continuous increase in the diagnostic performance over 250 training epochs; thus, almost no overfitting was observed (Fig. 1). The inclusion of multiple image classes from ImageNet (Fig. 3) during the training process may have supported the robustness and generalizability of the model. This led to the fact that only the desired dental findings became detectable instead of mistakenly interpreting similar pixel patterns on other image classes as dental defects (Fig. 2 and Tables 2 and 3). When discussing the potential limitations of this study, the image dataset has to be considered first. At the present stage, it can be assumed that the diagnostic performance might be equal in populations that are similar to those in the dataset, e.g., Caucasian children, adolescents and adults. In contrast, the evaluation of teeth from other ethnic populations or regions might possibly be lacking due to the known differences in the clinical appearance of teeth. Therefore, it would be essential to conduct external validation studies aimed at assessing the model performance independently from the used dataset. Furthermore, not all types of dental restoration or developmental or genetically determined disorders that affect teeth have been included in the model thus far. Consequently, the dataset and model need to be extended steadily to cover the spectrum of prevalent and rare dental pathologies as well as restorations as best as possible. Another limitation seems to be that the dataset consists of only high-quality dental photographs. Considering that images captured by various intraoral cameras, semiprofessional cameras or even mobile phones can also potentially be analyzed by the developed algorithm, the importance of proper image quality needs to be highlighted. This includes not only technical properties, e.g., correctly exposed und uncompressed images with an appropriate high resolution but also the ideal photographic representation of the object of interest. Therefore, it seems to be important, first, to safeguard high photographic image quality and, second, to include suboptimal images in future training sets. Such aspects require additional research. These technical aspects are also of importance and may influence and potentially limit the automatized feedback by the segmentation model when uploading own images of low quality.

In conclusion, the present diagnostic study demonstrated excellent model performance in detecting and localizing different caries and MIH classes from professional dental photographs. The study aim was reached by using a large image set with precise object annotations, image augmentation, and a transformer network. Nevertheless, the model needs to be further improved and evaluated under clinical conditions.

## Methods

### Ethical approval and reporting

This study on caries detection by AI-based methods was approved by the Ethics Committee of the Medical Faculty of the Ludwig-Maximilians University of Munich (project number 020-798). This study used anonymized intraoral photographs from earlier conducted investigations or from clinical situations in which images were taken for educational purposes. With respect to this, we were unable to identify any patients, and therefore, no written informed consent was possible. This investigation was reported following the recommendations of the Standards for Reporting of Diagnostic Accuracy Studies (STARD) steering committee^20^ and recently published recommendations for designing and conducting studies using AI methods in dental research^21^.

### Digital dental photographs

All clinical photographs were taken using standard procedures by experienced dentists (JK, RHW) over a period of more than ten years. In brief, clinical image acquisition included the use of professional single reflex lens cameras (Nikon D200, D300, D7100 or D7200, Nikon, Tokyo, Japan) equipped with a macro lens (Nikon AF-S Micro Nikkor 105 mm 1:2.8 G, Nikon, Tokyo, Japan) and a macro flash (EM-140DG, Sigma, Rödermark, Germany) after tooth cleaning and drying. Posterior teeth were photographed indirectly using intraoral mirrors^10,14,22,23^.

All available dental photographs from occlusal and freely accessible surfaces were processed anonymously. Aiming at safeguarding high image quality in the whole image set, insufficient photographs, e.g., over/underexposed, distorted or blurred images, were excluded. All included single tooth photographs were standardized according to the following parameters: aspect ratio of 1:1, resolution of 1200 × 1200 pixels with no compression, jpeg format and RGB color space. Thus, most of the included images were cropped and/or rotated by use of professional photo editing software (Affinity Photo, Serif, Nottingham, UK) until the tooth surface filled most of the frame. The dental image set included a broad spectrum of teeth that ranged from healthy to severely destroyed due to caries and MIH. Photographs with dental restorations, sealants, orthodontic appliances, or teeth with rare dental diseases, e.g., amelogenesis imperfecta or dentinogenesis imperfecta, were not excluded from the dataset. Finally, the image set comprised 18,179 single tooth photographs (4483 primary and 7699 permanent posterior teeth; 2339 primary and 3658 permanent anterior teeth). This sample represented the largest available number of single tooth photographs, which were further completed with high-quality annotations aiming at increasing the model performance.

### Dental pathology annotation (reference standard)

The anonymized image set was stored and processed on a university-based computer cloud to enable pixelwise labeling with the open source, web-based Computer Vision Annotation Tool (CVAT, server version 2.0, core version 4.2.1, Intel, Santa Clara, CA, USA). Initially, all images were split into five equal subsamples and were annotated by five trained and calibrated dental graduates (M.F., A.S., P.E., J.S., F.Z.). In case of questionable findings regarding detection, classification and size, these images or pathologies were re-examined and discussed with the experienced dentist (J.K., >20 years of clinical practice and scientific experience) until consensus on each diagnostic decision was reached. In another cycle, all annotations in terms of classifications and marked areas were independently checked and—if necessary—corrected by an experienced dentist (J.K.) with the aim of ruling out potential errors or misclassifications. The detection and classification of caries and MIH was made in agreement with widely accepted diagnostic scoring systems^24–29^. In detail, when a caries lesion was visually detectable in a clinical image, its location was annotated and classified according to the following scores: 1—non-cavitated caries lesion (first sign and established lesion), 2—grayish translucency, 3—localized enamel breakdown, 4—caries-related cavitation (dentin exposure and large cavity) and 5—largely/severely destroyed tooth with almost complete loss of the crown^24–28^. The following criteria were applied for chalky teeth detection: 1—demarcated opacity (hypomineralization/chalky tooth area with intact tooth surface), 2—enamel disintegration (hypomineralized hard tissue with enamel breakdown or dentin exposure) and 3—MIH-related restoration^29^. Each single tooth photograph could have multiple diagnostic findings (Table 1), which were annotated separately from each other. All dental annotations served as reference standards and were later used for cyclic training and evaluation of the transformer-based model.

Prior to the study, over the course of a 2-day workshop, all participating dentists were explicitly instructed in the field of dental diagnostics by the principal investigator (J.K.). The scoring reliability of all annotators regarding the detection and classification of caries and MIH was determined by diagnosing 140 single tooth photographs. The corresponding Kappa values for the intra- and inter-examiner reproducibility of the dental annotators (M.F., A.S., P.E., J.S., F.Z.) were found to be good to excellent for caries (intra: 0.858–1.000; inter: 0.656–0.837) and MIH (intra: 0.836–1.000; inter: 0.693–0.886). Permanent mutual exchange of knowledge between all annotators and the principal investigator was possible at any time during the study project. Furthermore, the dental work group had frequent and regular meetings to enable constant and proper decision making.

### Vision transformer-based model development (test method)

The AI-based algorithm for the detection, classification and localization of caries and MIH was trained using a pipeline of methods, mainly including image augmentation and the adaptation of a transformer network. Before training, the entire image set of single tooth photographs (*N* = 18,179 images) was randomly divided into a training set (*N* = 16,679), validation set (*N* = 500) and test set (*N* = 1000). With respect to the large image set, a test sample size of 1000 photographs with 1993 annotations (Table 1) was justified as appropriate to enable extensive model training and rigorous evaluation. The test set was not made available to the machine learning model as training material; it only served as an independent test set. The detailed composition of the image set in relation to registered pathologies is shown in Table 1.

Machine learning models require a large and variable number of training images to achieve excellent diagnostic performance. In this project, the dental image set was augmented with images from the open source ImageNet collection (https://image-net.org) containing 1,281,167 images in 1000 object classes by using Python (version 3.8.5, https://www.python.org), Pillow (PIL fork version 9.2.0, https://pillow.readthedocs.io) and AutoMould (git commit ca2bc76, https://github.com/UjjwalSaxena/Automold--Road-Augmentation-Library). In detail, a randomly selected ImageNet image was placed in the background of a newly generated image and combined with one to four randomly selected single tooth photographs in the foreground. Both the number of dental images and their placement over the background were random. The dental images were laid over the background, but no overlap of dental photographs was allowed. Each single tooth photograph was then randomly resized (from 512 × 512 to 1024 × 1024 pixels), rotated (0–360°), changed in terms of perspective (scaled up to 10% of the image size), randomly distorted and sharpened. This procedure resulted in a compiled image (RGBA format) that was further processed by random use of different image adjustments and filters: color (randomizing brightness, contrast, saturation, and/or color value), random motion blur (simulating camera shake during image acquisition), ISO noise (mimicking image noise), fog filter (faking fog and/or streaks in the image), sun flare (imitating overexposure) and image compression (simulating quality loss). This process of image augmentation resulted in a unique, randomly generated, virtual image (from 400 × 400 to 1200 × 1200 pixels in RGB format) that included all dental annotations (Fig. 3).

For the development of the machine learning model, a pretrained vision transformer encoder network with a multilayer perceptron decoder (SegFormer-B5, Nvidia, Santa Clara, CA, USA)^15^ was applied by utilizing an open source machine learning framework (PyTorch, version 1.12.0; https://pytorch.org/). Aiming for efficient neural network training, we used ZeRO-Offloading in the DeepSpeed library (Microsoft, Richmond, USA; https://www.deepspeed.ai/) and a decayed learning rate scheduler in our approach. The latter helped the model adjust pretrained knowledge over an initial warm-up phase of 1000 steps and assisted in monitoring and controlling for overfitting later. Furthermore, all virtual images were converted to brain floating point 16 (BF16) format. Thus, the amount of data to be processed per device was increased, whereby a technical batch size of *N* = 16 virtual images was achieved. ZeRO-powered data parallelism (ZeRO-DP) allowed the inclusion of eight servers, each equipped with a professional graphics card (RTX A6000 with 48 GB, Nvidia, Santa Clara, CA, USA), to work synchronously, increasing the actual batch size to *N* = 128. The machine learning model was trained over 250 epochs, which required an overall computing time of 7 days and 53 min. For this study, ~4.3 million different augmented images were virtually generated and made available to the machine learning model. In the final step, the model was finetuned by inputting all original and non-augmented dental images from the training set over five epochs.

### Statistical analysis

All images, including their annotations, were taken from the above described sample, and the model metrics were analyzed blindly (O.M.) by the dental work group using Python (version 3.8.5, http://www.python.org). Aiming at determining the AI model’s performance in localizing caries and MIH at the pixel level, the intersection over union (IoU), average precision (AP), F1-score, and accuracy (ACC) were calculated separately after 250 epochs of baseline training and finetuning. The IoU describes the overlap of the AI-predicted annotation with the control annotation—in our case, on a pixel level. IoU values above 0.5 can be considered good^30^. The AP indicates how precise a segmentation model is, i.e., how often the AI algorithm is correct in its prediction. To calculate the F1-score, the recall must first be determined. The recall describes how good the segmentation model is at making positive predictions. The F1-score is the mean value of AP and recall. The ACC is the fraction of all predictions that the AI model predicted correctly. All values were determined separately for all caries and MIH classes. The overall diagnostic accuracy (ACC = (TN + TP)/(TN + TP + FN + FP)) was determined by calculating the number of true positives (TP), true negatives (TN), false positives (FP) and false negatives (FN) when at least one pixel was identified in the corresponding category per image. Consequently, the sensitivity (SE), specificity (SP), positive and negative predictive values (PPV and NPV, respectively) were computed.

### Reporting summary

Further information on research design is available in the Nature Research Reporting Summary linked to this article.

### Supplementary information

Reporting Summary

## Data Availability

Access to the model data or the annotated dataset can be made on reasonable request. The developed transformer model is openly accessible as a web application. Please visit http://demo.dental-ai.de.
